# Disease-associated metabolic pathways affected by heavy metals and metalloid

**DOI:** 10.1016/j.toxrep.2023.04.010

**Published:** 2023-04-24

**Authors:** Zinia Haidar, Kaniz Fatema, Sabrina Samad Shoily, Abu Ashfaqur Sajib

**Affiliations:** Department of Genetic Engineering & Biotechnology, University of Dhaka, Dhaka 1000, Bangladesh

**Keywords:** Heavy metal, Metalloid, Disease-associated metabolic pathways, Arsenic, Cadmium, Chromium, Iron, Mercury, Nickel, Vanadium

## Abstract

Increased exposure to environmental heavy metals and metalloids and their associated toxicities has become a major threat to human health. Hence, the association of these metals and metalloids with chronic, age-related metabolic disorders has gained much interest. The underlying molecular mechanisms that mediate these effects are often complex and incompletely understood. In this review, we summarize the currently known disease-associated metabolic and signaling pathways that are altered following different heavy metals and metalloids exposure, alongside a brief summary of the mechanisms of their impacts. The main focus of this study is to explore how these affected pathways are associated with chronic multifactorial diseases including diabetes, cardiovascular diseases, cancer, neurodegeneration, inflammation, and allergic responses upon exposure to arsenic (As), cadmium (Cd), chromium (Cr), iron (Fe), mercury (Hg), nickel (Ni), and vanadium (V). Although there is considerable overlap among the different heavy metals and metalloids-affected cellular pathways, these affect distinct metabolic pathways as well. The common pathways may be explored further to find common targets for treatment of the associated pathologic conditions.

## Highlights

• Heavy metal-induced oxidative stress is damaging for biomolecules such as DNA, proteins, and lipids.
• Exposure to heavy metals affects cellular pathways associated with various disease conditions.
• Some of the metals may play a protective role against pathological conditions like diabetes and neurodegeneration.

## Graphical Abstract

**Figure:**
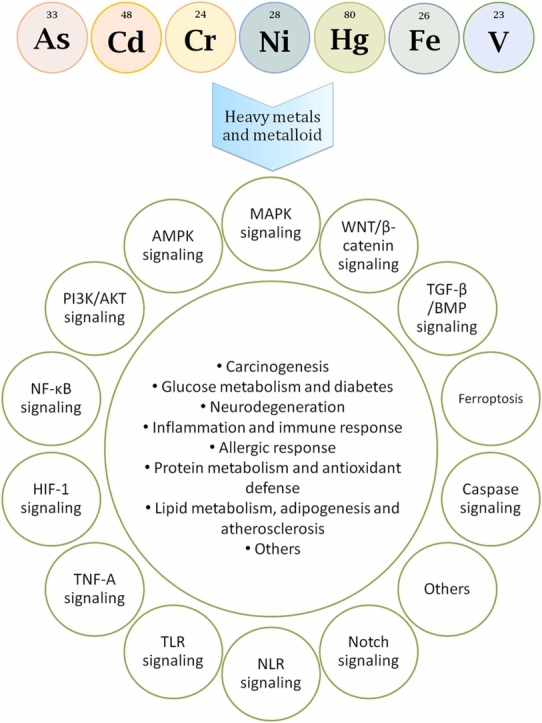

## 1 Introduction

Heavy metals and metalloids have atomic numbers > 20 and densities > 5 g/cm^3^ [1]. Some essential heavy metals including chromium (Cr), cobalt (Co), copper (Cu), iron (Fe), manganese (Mn), molybdenum (Mo), selenium (Se), and zinc (Zn) have vital biochemical and physiological roles in animals and plants at low concentrations [2], [3]. These trace metals are significant constituents of some critical enzymes involved in redox reactions, biosynthesis, transport, and other metabolic activities [4]. Non-essential heavy metals and metalloid like arsenic (As), cadmium (Cd), mercury (Hg), nickel (Ni), and vanadium (V) have no known essential biological purpose; rather, they exert adverse health effects [4], [5]. However, both essential and non-essential heavy metals can become toxic if their concentrations exceed certain thresholds [2]. Due to the intricate nature of the interactions between heavy metals and biological systems, the growing incidences of exposure to these elements have become increasingly challenging and difficult to address globally [6]. According to the International Agency for Research on Cancer (IARC), arsenic, hexavalent chromium, cadmium, and nickel are classified as group 1 carcinogen [7]. Arsenic and cadmium exert deleterious effects on glucose metabolism and other metabolic pathways. Glucose homeostasis has also been reported to be affected by manganese, mercury, nickel, and zinc [8]. Exposure to arsenic, cadmium, copper, nickel, lead, and zinc increases the risk of developing diabetes [9]. Potential link has been suggested between heavy metal exposure and cardiovascular complications. Such cardiotoxic heavy metals include arsenic, cadmium, lead, and mercury. Imbalances in essential metals, including copper, manganese, nickel, and zinc, are also associated with an increased risk of cardiovascular diseases (CVDs) [6], [10]. Arsenic, cadmium, lead, and mercury are among the known endocrine disruptors that can affect brain development in the fetus as well as infant growth [11]. An increased risk for the onset and progression of neurodegenerative diseases, such as Alzheimer’s disease, Huntington’s disease, Parkinson disease, muscular dystrophy, and multiple sclerosis, was demonstrated following exposure to heavy metals, including arsenic, cadmium, copper, iron, lead, manganese, and mercury [11], [12].

Humans can be occupationally and unintentionally exposed to heavy metals and metalloids [13], [14]. Anthropogenic activities including urbanization and industrialization have increased human exposure to these heavy metals [15]. Many occupations involve exposure to these metals and their conjugates [14]. Mining and smelting, use of fertilizers and pesticides, land application of wastewater and sewage sludge, electronic device disposal, and fossil fuel burning are some of the anthropogenic causes responsible for heavy metal exposure [5], [13], [16]. While ingestion or skin absorption of heavy metals is more common in general, inhalation of heavy metals is often the case in occupational settling. Vast numbers of workers are co-exposed to cadmium, cobalt, lead, and nickel in all industrial countries. Occupational exposure is mostly caused by industries that make chemical stabilizers and metal coatings, metal alloys, batteries, plastics, textiles, microelectronics, paint, wood preservatives, cosmetics, herbicides, pesticides as well as nuclear power plants. The elements used in such industrial plants are often released into the air during combustion or into the soil or water bodies as effluents [4], [13]. However, the sources of heavy metals in the environment can be natural as well [16]. Many of these heavy metals occur naturally in the earth's crust. Natural processes like volcanic eruptions, spring waters, erosion, sediment resuspension, and bacterial activity deposit these metals in soil and water systems [4], [13].

These metals are bioaccumulative in nature [1]. Heavy metals are absorbed by plant roots and leaves and accumulated in fruits and vegetables. Contaminated fish, shellfish, and seafood can cause heavy metal poisoning [17]. In fact, the primary source of exposure to toxic heavy metals such as cadmium, lead, mercury, or nickel for individuals who are not professionally exposed is through the consumption of contaminated foods. Cereals, vegetables, meat and fish are the major contributors of dietary cadmium intake. In case of lead, water and beverages are the major contributors followed by vegetables, meat and meat products, milk and dairy products, and cereals. The intake of methylmercury (MeHg) is strongly associated with the quantity of fish consumed. Other contributors of dietary mercury intake include cereals, vegetables, and milk products. Nickel is mainly consumed through cereals, vegetables, sugars, water and beverages, and fruits [18]. Vegetables, which are rich sources of important nutrients and antioxidants, are widely consumed by people all around the world. However, as these absorb both essential and toxic metals through the contaminated soil, consumption of metal-contaminated vegetables has been linked to a range of human health concerns including cancers. Amaranth, coriander, eggplant, spinach, and tomato were found to contain high concentrations of heavy metals like cadmium, chromium, copper, iron, lead, manganese, mercury, nickel, and zinc [19]. Continuous monitoring of heavy metals in commercialized edible fish products, both freshwater (carp, flounder, rainbow trout, tench, tilapia, perch, blue grenadier, gilthead seabream, mackerel, etc.) and marine (eel, false kelpfish, croakers, etc.) is strongly recommended to avoid consumption of excess levels of arsenic, cadmium, chromium, copper, iron, lead, mercury, and zinc [20], [21], [22].

**Fig. 1:**
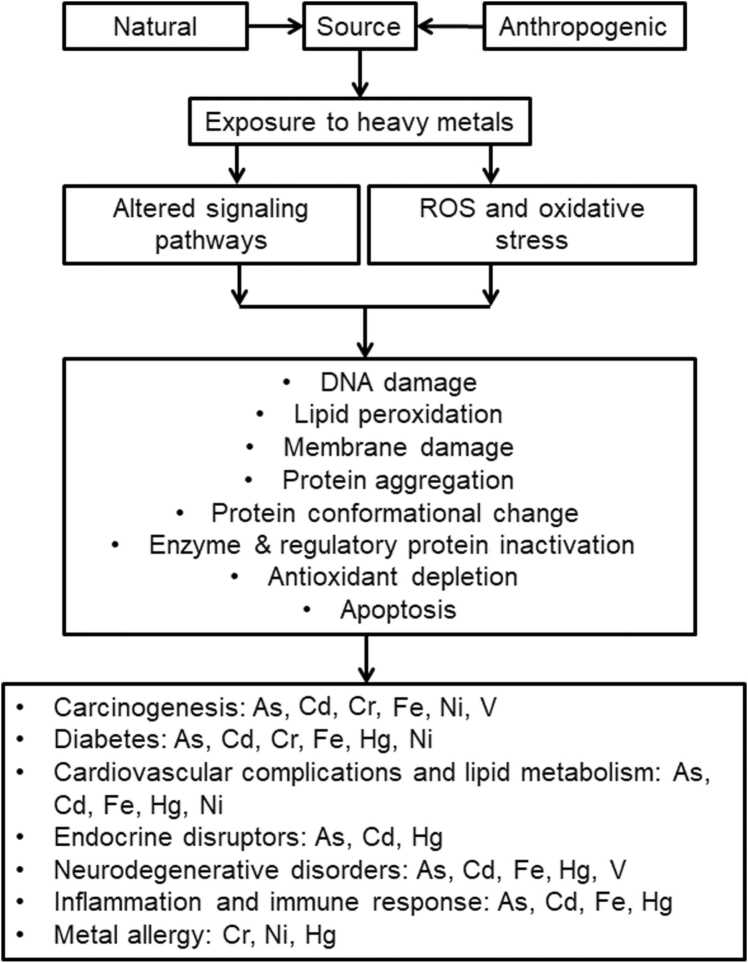
Sources of exposure to heavy metals and mechanisms of heavy metal toxicity.

Heavy metals and metalloids trigger cell signaling cascades [13]. These signaling pathways and their regulatory components regulate cell growth, proliferation, differentiation, cell cycle regulation, DNA repair, immunological response, malignant transformation, and apoptosis, among others ( Fig. 1). Some of these heavy metals and metalloids, including arsenic, cadmium, chromium, iron, mercury, nickel, and vanadium, have become a significant public health concern due to their highly toxic nature among others [4], [23], [24], [25]. This study investigates how these seven metalloid and metals (As, Cd, Cr, Fe, Hg, Ni, and V) affect cellular pathways and are associated with chronic diseases such as diabetes, cardiovascular diseases, carcinogenesis, neurodegenerative diseases, endocrine and reproductive abnormalities, inflammation, and allergic reactions.

## 2 Common molecular mechanisms of heavy metal toxicity

Toxic effects from a heavy metal depend on the type of metal, its chemical properties (i.e. type of metal conjugates and oxidative state), dose, duration, route of exposure, interaction with other chemicals in the environment, and the exposed individual's age, gender, genetics, disease states, nutritional and immunological status [4], [26]. In the human body, heavy metals are transported into cells, tissues and organs, where their interaction with biomolecules, including DNA and enzymes, disrupts cellular, endocrine, immunological, neurological, and reproductive functions [4], [13], [16] ( Table 1). The majority of cellular disruptions are caused by metals forming stable complexes with enzymes and receptors, thus blocking them, or by the production of reactive oxygen species (ROS), which disrupt the cell's oxidative environment [3], [27]. The generation of free radicals in cells by ionic heavy metals results in oxidative damage [3]. ROS are generated and consumed as part of normal metabolism. However, imbalance in their homeostasis and loss of control of their management are involved in the pathogenesis and the progression of different human diseases [28]. The Fenton reaction is one of the most common routes through which heavy metals such as iron, chromium, and vanadium produce hydroxyl radicals in the presence of hydrogen peroxide [13], [29]. ROS can cause DNA damage as well as DNA strand breaks, *i.e*., single strand breaks (SSBs) and double strand breaks (DSBs), protein-protein cross-link formation, polypeptide backbone oxidation, amino acid side chain oxidation (particularly cysteine), and lipid peroxidation [30]. Heavy metals exert toxicity at protein level in a pleiotropic manner. These metals can bind and displace the original metal from proteins or metalloenzymes, causing cell dysfunction and toxicity [23]. These mostly interact with the —SH and —NH2 groups of proteins, altering conformations and inactivating enzymes [4]. Depletion and inhibition of enzymes like glutathione (GSH) reductase induces ROS buildup and oxidative damage [4], [31].

**Table 1:** Prominent metabolic effects of As, Cd, Cr, Fe, Hg, Ni and V.

Heavy metals/ metalloid	Effects
Arsenic	• Increased risk of type 2 diabetes and a significant increase in morbidity and mortality compared to unexposed area [32], [33]. • Cardiovascular diseases (CVDs) e.g., atherosclerosis, coronary artery disease, and hypertension [34]. • Respiratory diseases e.g., pulmonary tuberculosis, bronchitis, and lung cancer [35]. • Neurotoxicity e.g., neuropsychological, neural, and nervous dysfunctions including language learning, executive functioning, memory, processing speed, mental retardation, and developmental disabilities [36], [37]. • Renal dysfunctions including albuminuria, proteinuria, and chronic kidney disease (CKD) [38]. • Cancers of internal organs including bladder, kidney, liver, lung, uterus, and prostate [39]. • GI disturbances, including nausea, vomiting, abdominal pain, and diarrhea [24]. • Abnormal black-brown skin-pigmentation of the skin (melanosis) and skin lesions i.e. hardening of palms and soles or keratosis leading to hyperkeratosis and skin cancer [40]. • Serious social implications, including social instability, social discrimination, ostracism, and marriage related problems, especially in developing countries like Bangladesh [36].
Cadmium	• Diabetes and diabetes related kidney diseases [41]. • CVDs including hypertension, stroke, heart failure, atherosclerosis, and peripheral arterial disease [42]. • Respiratory complications including flu-like symptoms (chills, fever, etc.) followed by chest pain, cough, and dyspnea, bronchospasm and pulmonary edema, intra-alveolar hemorrhage, thrombosis, lung damage and inflammation, chronic rhinitis, bronchitis, even lung cancer [43], [44], [45]. • Neurological alterations such as headache, lower attention span, learning disorder, hyperactivity, olfactory dysfunction and memory deficits, even neuronal cell death and cell signaling pathway disturbances [46]. • Cancer of kidney and other renal dysfunctions such as nephrotoxicity and nephropathy, renal tubular and glomerular dysfunction, kidney stones, and renal failure manifested by aminoaciduria, glucosuria, hypercalciuria, hyperphosphaturia, polyuria, etc. [41], [47], [48], renal damage leading to bone lesions, reduced mineral density in bones and other effects of osteomalacia and osteoporosis [48]. • Cancers of vital organs like prostate, urinary bladder, breast, and liver [43].
Chromium	• Increased risk of CVDs [49]. • Sneezing, watery nasal discharge, labored breathing, choking sensation in the throat, asthma and other respiratory distresses like chronic bronchitis, chronic irritation, chronic pharyngitis, and chronic rhinitis [50], [51]. • Symptoms of dizziness, headache, and weakness [50]. • Renal toxicities including acute tubular necrosis, acute renal failure, low molecular weight proteinuria, and CKD [52], [53]. • Lung cancer and cancers of the nose and nasal sinuses [54], [55]. • Type IV hypersensitivity reaction characterized by eczema; GI hemorrhage, vertigo, thirst, abdominal pain, and bloody diarrhea; and in severe cases, coma and death [56].
Iron	• Insulin resistance, type 2 diabetes, and other conditions including gestational diabetes and prediabetes as well as obesity, MetS [57]. • CVDs and arterial thrombosis [57], [58]. • Respiratory and lung diseases including chronic obstructive pulmonary disease, lung cancer, cystic fibrosis, idiopathic pulmonary fibrosis and asthma [59]. • Neurological disorders e.g., epilepsy, Alzheimer’s disease, Parkinson’s disease, Huntington’s disease, and amyotrophic lateral sclerosis [60]. • Increased urinary iron excretion, renal iron deposition, kidney injury and kidney failure [61]. • Different types of carcinomas such as prostate cancer, pancreatic cancer, colorectal cancer, lung cancer, breast cancer, haematological cancers (leukaemias, lymphomas and myelomas such as multiple myeloma and non-Hodgkin’s lymphoma), head, neck, and renal cell carcinomas, and hepatocellular carcinoma [62], [63], [64].
Mercury	• Insulin resistance, obesity, and MetS [65]. • CVDs including dyslipidemia, hypertension and atherosclerosis [65], [66]. • Pulmonary conditions including bronchitis, pneumonitis, and pulmonary fibrosis [25], [67]. • Neurotoxicity that includes cognitive impairment, hearing loss, disequilibrium, constriction of the visual-field, memory problems, cerebellar ataxia, dysarthria, postural and action tremor [4], [68]. • Renal toxicity, especially to renal tubules [69]. • GI disturbances including abdominal pain, indigestion, inflammatory bowel disease, ulcers and bloody diarrhea [25]. • Endocrine disruption [70], [71]. • Reproductive toxicity [25].
Nickel	• Prevalence of type 2 diabetes [72]. • Increased risk of CVDs [73]. • Respiratory diseases including asthma and bronchitis, inflammations in the respiratory tract and lungs, and lung fibrosis [74], [75]. • Neurotoxicity, certain neurodegenerative and neuropsychiatric diseases, cognitive impairments, and mitochondrial dysfunctions [75], [76], [77]. • Acute renal impairments including CKD and bone disorders [78], [79]. • Cancer of the respiratory tract, lung cancer, and cancers of the nose and nasal sinuses [54], [75]. • Inflammation of the digestive tract, and symptoms of irritable bowel syndrome [80]. • Contact dermatitis and skin allergy [78]. • Instant symptoms such as nausea, vomiting, vertigo, and irritation followed by delayed type symptoms like stiffness of the chest, constant cough, palpitations, sweating, tachycardia, visual disturbances, and weakness [74].
Vanadium	• Increased risks of a variety of pathologies like hypertension, CNS damage and neurological disorders, bronchial hyper-reactivity, systemic inflammation, hyper-coagulation, and cancers [81], [82].

## 3 Metabolic pathways affected by heavy metals and metalloid

### 3.1 Carcinogenesis

#### 3.1.1 Effects of arsenic

The IARC classifies arsenic as a group 1 carcinogen [83]. When ingested, arsenic enters the cell predominantly in its pentavalent ‘arsenate’ form (As[V]). Once inside, As[V] is reduced to a more toxic trivalent ‘arsenite’ form (As[III]) that further undergoes hepatic methylation to form mono- and dimethyl arsenical species (MMA and DMA, respectively) by arsenite methyltransferase (AS3MT) [84]. The generation of MMA and DMA causes GSH depletion and ROS production.

Inorganic arsenic is known to induce sister chromatid exchange and intrachomosomal homologous recombination. Enhanced recombination frequency may be related to inhibition of DNA replication and subsequent strand breaks [85]. Arsenic activates the phosphatidylinositol 3-kinase/protein kinase B (PI3K/AKT) signaling pathway through increasing the activity of PI3K and subsequent phosphorylation of AKT through ROS generation, along with a possible role of mitogen-activated protein kinase (MAPK) signaling. Activation of the PI3K/AKT pathway promotes the mechanistic target of rapamycin (mTOR), effectors of which include hypoxia-inducible factor 1 (HIF-1), activator protein 1 (AP-1), and nuclear factor kappa B (NF-κB) that play an important role in promoting cancer. Increased pathway activity and significant elevation in the levels of PI3K, AKT, and mTOR was observed in studies conducted on cell lines although there were wide variations in dose (0 to 20 μM) and duration of treatment (4 hours to 26 weeks) [86]. The PI3K/AKT signaling network has numerous metabolic consequences. Aberrant activation of this pathway is one of the most frequent events in human carcinogenesis that results in uncontrolled cell growth, survival, and metabolism [87]. Arsenic-induced cell proliferation, migration and invasion ability, angiogenesis, and chemoresistance may be dependent on PI3K/AKT signaling [88], [89]. Arsenic-induced increased expression of HIF-1α through PI3K/AKT activation can also upregulate vascular endothelial growth factor (VEGF) and promote malignant transformation and proliferation [90]. Elevated HIF-1 correlates with tumor metastasis, angiogenesis, and poor prognosis [91].

Arsenic-induced carcinogenesis involves members of the MAPK families. Low-level (1 to 2 µM) arsenic trioxide (ATO) exposure activates extracellular signal-regulated kinases (ERK1/2), causing cell transformation [92], [93]. Inhibition of such activation enhances arsenic-induced apoptosis in acute promyelocytic leukemia (APL) cells [92]. Alternatively, high concentrations (> 50 to 200 µM) of arsenite induce apoptosis by the activation of c-Jun N-terminal kinase (JNK) and NF-κB pathways, suggesting a chemotherapeutic role of arsenic, which is already utilized in the treatment of APL [93]. Inhibition of JNK suppresses arsenic-dependent apoptosis [94]. ATO-induced ROS and intracellular redox imbalance stimulate the p38 MAPK pathway and induce apoptosis via the caspase-3 pathway, establishing the role of ATO in anticancer treatments for APL and chronic myelogenous leukemia (CML) [95]. The role of p38 activation in apoptosis induction is not clear. One study found that p38 inhibitors decreased ATO-dependent apoptosis, suggesting a function for p38 activation in apoptotic cell death [95]. However, another study found that p38 is a negative regulator of ATO-induced apoptosis [94]. The latter proposed that inhibition of p38 activation enhances ATO-dependent JNK kinase activity, which is required for apoptosis. ATO and PI3K/mTOR inhibitors are being combined to treat APL and breast cancer [86], [96]. Arsenic-associated skin carcinogenesis involves MAPK, NF-κB, and keratinocyte growth factor modulation [39].

#### 3.1.2 Effects of cadmium

The half-life of cadmium is 25 to 30 years [97]. According to the IARC, cadmium is a group 1 human carcinogen [98]. Cadmium produces ROS and depletes GSH [98], [99]. Cadmium causes lung, breast, prostate, pancreatic, urinary bladder, kidney, and nasopharynx cancers [97], [100]. The postulated mechanism behind cadmium-associated malignancies is a cascade of events beginning with tumor necrosis factor α (TNF-α) and nuclear factor-erythroid factor 2-related factor 2 (Nrf2) overexpression, followed by ROS production and aberrant gene expression, dysregulation of cell proliferation, and apoptosis resistance [15]. Similar to arsenic, cadmium causes time- and dose-dependent malignant transformation via HIF-1α (significant induction at 1.25, 2.5, and 5µM CdCl_2_ but attenuation at (10 and 20 µM) VEGF (induced by 5 µM CdCl_2_) overexpression via ERK and PI3K/AKT pathways (induced by 5, 10, and 20 µM CdCl_2_) and ROS generation [90], [101]. Cadmium-induced ROS production in mitochondria activates the NF-κB pathway, increasing HIF-1α expression and worsening lung damage through macrophage activation [102]. Low-dose (1 µM) cadmium activates the p21-dependent MAPK pathways, including ERK1 and p38, but not the PI3K pathway, along with the activation of *c-fos* and *c-myc* early genes and NF-κB signaling-dependent genes, and promotes macrophage proliferation [103]. Long-term (10 or 20 µM CdCl_2_ for 9 to 15 weeks) cadmium exposure can cause lung cancer via activating the Notch1 signaling system. HIF-1α, AKT, and ERK may activate Notch1 in this mechanism as found in cell culture studies [104]. Cadmium-induced renal carcinogenesis may be caused by an altered β-catenin signaling pathway [15].

#### 3.1.3 Effects of chromium

Hexavalent chromium Cr[VI] compounds are highly toxic and cause most chromium toxicity. Cr[VI] has been classified as a human pulmonary carcinogen [56], [105], whereas trivalent Cr[III] is essential for humans and animals, playing a role in glucose, fat, and protein metabolism [4], [23].

Several factors are involved in chromium-induced carcinogenicity, including tissue and cell type, Cr[VI] concentration, exposure period, free radical formation, and Cr[V] and Cr[IV] reactivity. The hexavalent Cr[VI] does not bind to DNA or other macromolecules itself; rather, it is reduced to Cr[V], Cr[IV], and Cr[III] by reacting with cellular reductants, triggering Fenton-type reactions. This generates hydroxyl radicals in the presence of hydrogen peroxide and induces oxidative stress. Chromium cross-linking and adduct formation with cellular antioxidants cause oxidative stress and ROS generation leading to DNA damage, including DNA–chromium–protein crosslinks, DNA inter- and/or intra-strand crosslinks, SSBs and DSBs, p53 point mutations, and lipid peroxidation [15]. These DNA–chromium adducts are difficult to repair and cause cellular malignancy. Hexavalent Cr[VI] activates the nuclear factor-erythroid factor 2-related factor 2/Kelch-like ECH-associated protein 1 (Nrf-2/Keap1) signaling pathway that plays a protective role by reducing chromium-induced ROS and apoptosis. In contrast to Nrf null cells, which could produce ROS at concentrations as low as 10 µM, wild-type cells needed higher doses of Cr[VI] (> 50 µM) to promote ROS production [106]. Aberrant overactivation of the Nrf-2 pathway increases cancer cell proliferation by metabolic reprogramming, inhibition of cancer cell apoptosis, and augmentation of cancer stem cell self-renewal potential, indicating a bad prognosis [107]. Chromium activates the PI3K/AKT signaling pathway, however its role in chromium-induced carcinogenesis is unknown [89]. Co-exposure to Cr[VI] and Cr[III] promotes malignant cell transformation via ERK and AKT signaling pathways [108]. Chronic Cr (VI) exposure has been shown to impair immune function in Wistar rats [109].

#### 3.1.4 Effects of iron

Iron is found in the earth’s crust and coexists with manganese in groundwater [14], [110]. Iron overload causes free radical generation [30]. Iron metabolism and cancer biology are interconnected. Iron overload causes malignant transformation, neoplastic cell proliferation, immunological evasion, and therapeutic resistance [111], [112]. On the other hand, ferroptosis caused by elevated levels of iron or by iron-chelating agents both have anti-cancer potential [112]. Iron is essential for cell survival, especially in highly active cells like tumor cells, since DNA replication is iron-dependent [113], [114]. Iron-induced carcinogenesis may be linked to iron homeostasis disruption and ROS elevation. HIF activation increases iron absorption in tumor cells [64]. In human head and neck squamous carcinoma cells, iron overload activates ERK1/2 and AKT signaling pathways and upregulates matrix metalloproteinase-9 (MMP-9) in a dose-dependent manner (significant responses at 15 and 25 µg/ml of ferric ammonium citrate). MMP-9 was found to be associated with the invasive and metastatic properties in cell line studies [115]. Bone morphogenetic proteins (BMPs) sense the cell's iron state. High systemic iron enhances hepcidin expression [113], [116]. High BMP levels coupled to increased hepcidin expression are connected to multiple myeloma, non-Hodgkin’s lymphoma, prostate, lung, breast, and renal carcinoma [63]. Compared to systemic iron, dietary iron augments wingless/integrated (WNT) signaling. Iron-induced malignant progression, including colorectal cancer, involves aberrant WNT signaling and β-catenin buildup [64]. WNT/β-catenin signaling system, similar to TGF-β pathway, regulates cancer cell metastasis and invasion, and iron modulates both routes [112]. Ferritin's close association with the NF-κB signaling pathway can contribute to iron-induced inflammation and carcinogenesis [64]. Excessive iron impairs the function of CD4^+^ lymphocytes crucial for anti-tumor activity [117], [118]. Drugs, medications, and other chemical compounds that induce ferroptosis in different cancer cells, such as pancreatic cancer, hepatocellular carcinoma, gastric cancer, and colorectal cancer, among others, are well-established [119].

#### 3.1.5 Effects of vanadium

Vanadium is a metalloid with different oxidation states that can generate free radicals [13], [82]. The pentavalent ‘vanadate’ is the most toxic and is readily taken up by erythrocytes [120]. Vanadium-induced ROS production and oxidative stress cause lungs or lung-associated cell apoptosis among other effects [81]. Vanadate-induced ROS and HIF-1α and VEGF expression through the PI3K/AKT pathway in human prostate carcinoma cells may contribute to vanadate-induced carcinogenesis. HIF-1α was induced by vanadate at a dose-dependent manner with maximum expression induced by 100 µM vanadate after 6 hours of treatment [121]. Vanadate-generated ROS also stimulates the activation of MAPK pathways—p38 and ERK, which can upregulate p21 and arrest cell growth [122]. Vanadate-induced DNA damage also increases p53 activity. Vanadate-damaged cells undergo apoptosis due to ROS generation and p53 activation [123]. However, those destined for apoptosis sometimes escape and may be responsible for vanadate-induced carcinogenesis [124].

Vanadium complexes have anti-cancer properties, too, including generation of ROS, inhibition of tyrosine phosphatases, induction of apoptosis, DNA cleavage, cell cycle arrest, and lipoperoxidation [125], [126]. One anti-proliferative vanadate compound was discovered to induce apoptosis in breast cancer cells by activating caspase-3, inhibiting Notch signaling, and arresting cell cycle [127]. Some vanadium compounds are experimentally able to counteract tumor metastasis [128]. Most of these studies were conducted on cell lines rather than in animal models.

#### 3.1.6 Effects of other heavy metals

Nickel, a group 1 carcinogen, depletes cellular GSH and increases ROS [75], [129]. Nickel nanoparticles activate HIF-1, promoting cell transformation and tumor progression [130]. Nickel enhances pathophysiological angiogenesis by producing VEGF through AKT, ERK, and NF-κB activation. Uncontrolled angiogenesis may contribute to nickel-induced carcinogenicity, including respiratory tract and lung cancer [131]. On the other hand, carcinogenicity of mercury, a group 3 carcinogen meaning “not classifiable as to their carcinogenicity to humans,” is inconclusive. It is naturally present in three forms: elemental, inorganic, and organic, each with its own type of toxicity. MeHg is the most frequently encountered organic form in the environment, generated by microbial activity [4]. Due to its lipophilic nature, MeHg can easily cross the placental and blood–brain barriers, affecting the developing fetal brain and nervous system [4], [14], [132]. Organic and inorganic mercury increases the production of ROS [4]. Mercury can be an epigenetic carcinogen since it can impair gap junction intercellular communication and cause immunosuppression, according to one study [133]. Mercury activates stress genes involved in cell cycle regulation and apoptosis in human liver carcinoma cells, according to lab tests [67]. Table 2 summarizes the major carcinogenesis-associated signaling pathways that are affected by heavy metals and metalloids.

**Table 2:** Crucial signaling pathways associated with carcinogenesis that are affected by heavy metals and metalloids.

Heavy metals and metalloids	Key signaling pathways associated with carcinogenesis^a^
Arsenic	• PI3K/AKT [86] • NF-κB [93] • JNK [93] • p38 [95] • ERK [92] • HIF-1α [90]
Cadmium	• PI3K/AKT [101] • ERK [101], [103] • HIF-1α [101], [102] • NF-κB [102] • p38 [103] • Notch [104] • WNT/β-catenin [15] • TNF-α [15] • Nrf-2 [15]
Chromium	• PI3K/AKT [108] • ERK [108] • Nrf-2 [106]
Iron	• AKT [115] • ERK [115] • NF-κB [64] • HIF-1α [64] • WNT/β-catenin [64] • BMP [63]
Mercury	• Potential epigenetic carcinogen [133]
Nickel	• PI3K/AKT [131] • NF-κB [131] • ERK [131] • AMPK [131] • HIF-1α [130]
Vanadium	• PI3K/AKT [121] • HIF-1α [121] • p38 [122] • ERK [122] • p53 [123] • Caspase-3 [127] • Notch [127]

a other than direct reactive oxygen species (ROS) generation

### 3.2 Glucose metabolism and diabetes

#### 3.2.1 Effects of arsenic

Insulin resistance causes type 2 diabetes (T2D) [134], [135]. Peroxisome proliferator-activated receptor γ (PPARγ) and AKT play crucial roles in glucose metabolism [135], [136]. PPARγ increases the expression of insulin-sensitive genes including glucose transporters type 2 (GLUT2), type 4 (GLUT4), and β-glucokinase [134], [135], [136]. Trivalent form of iAs (As[III]) inhibits PPARγ and mTOR Complex 2 (mTORC2)-target PKB/AKT in liver cells and adipocytes [33], [137], [138]. By suppressing expression and phosphorylation of AKT, iAs interferes with GLUT4 mobilization (0.5 to 1 mM of arsenite or 5 to 30 µM of phenylarsine oxide), hence inhibits glucose uptake in adipocytes [33], [139], and can even trigger GLUT4 degradation in adipocytes [140], [141]. Insulin-stimulated p38 MAPK phosphorylation boosts GLUT4 translocation. Arsenic can also alter p38 MAPK signaling and insulin-stimulated glucose absorption, leading to insulin resistance and T2D [141], [142]. Chronic arsenic exposure increases TNF-α (at a concentration of 1 µM) and interleukin 6 (IL-6), which cause insulin resistance. Low levels of arsenite (≤ 1 µM) also activate NF-κB that is linked with both insulin resistance and β cell dysfunction. However, higher concentrations (≥ 5 µM) promote apoptosis rather than NF-κB activation [139]. T2D disrupts pentose-glucuronate interconversion. Metabolites that are associated with iAs exposure are strongly correlated with this pathway [143], [144].

Arsenic compounds bind to —SH and—PO_4_ groups in glucose-metabolizing enzymes and biomolecules with high affinity. Pentavalent As[V] interacts with ATP phosphate binding sites and inhibits ATP-dependent processes like pentose phosphate pathway (PPP) and insulin secretion [139]. Trivalent As[III] forms covalent bonds with the disulfide bridges present in insulin, insulin receptors, GLUTs, and enzymes e.g*.*, pyruvate dehydrogenase and α-ketoglutarate dehydrogenase [139]. In its oxidative phase, the PPP creates reduced nicotinamide adenine dinucleotide phosphate (NADPH), an important GSH cofactor. Arsenic exposure significantly downregulates glucose-6-phosphate dehydrogenase (G6PDH), which catalyzes NADPH production. Disruption of the PPP pathway and reduced activity of G6PDH further interrupts the cell’s ability to deal with oxidative stress and can lead to oxidative stress-induced diabetes [145]. Arsenic-induced alteration in glucose absorption may also raise the risk for metabolic syndrome (MetS) because of a linear association between arsenic concentrations and MetS components, such as plasma glucose, lipids, and blood pressure [146]. MetS is a major risk factor for T2D and CVDs [147].

#### 3.2.2 Effects of cadmium

Cadmium affects insulin secretion from pancreatic β-cells. While low cadmium levels enhance insulin release, high levels diminish the rate [41]. Subchronic exposure (1.0 to 2.0 mg/kg cadmium for 7 to 14 days) significantly increases blood glucose levels by increasing the activities of all four gluconeogenesis enzymes: hepatic pyruvate carboxylase, phosphoenolpyruvate carboxykinase, fructose 1,6-bisphosphatase, and glucose-6-phosphatase [41], [148]. Cadmium lowers glucose transport and GLUT4 expression. Such interruptions may contribute to diabetes, diabetes-related hyperglycemia, and kidney diseases [41]. Cadmium-mediated PPARγ downregulation inhibits pre-adipocyte differentiation, and adipocyte differentiation failure has been linked to T2D [149], [150]. Decreased PPARγ also causes a decrease in adipose tissue mass and disruptions in adipose-derived hormones which may contribute to glucose and lipid dysregulation, insulin resistance, CVDs, and hypertension [151]. Cadmium-induced elevation of pro-inflammatory lipids such as lysophosphatidylcholine (lysoPC) can activate the inhibitory kappa B kinases (IKKs) that regulate the NF-κB pathway and is associated with chronic inflammatory diseases like obesity and diabetes. Cadmium exposure also elevates pro-inflammatory cytokines like TNF-α, IL-6 and IL-1β, which are linked to diabetes [152].

#### 3.2.3 Effects of chromium

Chromium, unlike arsenic and cadmium, may help patients with diabetes and MetS by improving insulin sensitivity and glucose metabolism [153]. Another study, however, revealed no significant effect of chromium on glucose or lipid metabolism in non-diabetics [154]. Chromium supplements reduce the risk of T2D [154], [155]. Chromium binds to insulin receptors and stimulates their tyrosine kinase activity, potentiating insulin action. It's a cofactor for optimum insulin activity [153], [154]. It also increases insulin sensitivity by inhibiting phosphotyrosine phosphatase that dephosphorylates insulin receptors [153]. Chromium stimulates insulin signaling downstream effectors, such as the PI3K/AKT pathway, which increases GLUT4 translocation activity and transiently enhances the AMP-activated protein kinase (AMPK) pathway, resulting in increased glucose uptake [156]. At the same time, chromium suppresses the tyrosine phosphorylation of c-Jun by inhibiting the JNK pathway. Phosphorylated c-Jun usually attenuates insulin signaling by phosphorylating the serine residue of insulin receptor substrate 1 (IRS-1) [156].

#### 3.2.4 Effects of iron

High levels of nonpathological iron, such as dietary iron, have been found to be associated with an increased risk of diabetes. Elevated serum ferritin levels are associated with insulin resistance, T2D (including gestational diabetes and prediabetes), obesity, MetS, and CVDs [57]. High ferritin levels shows a positive correlation with MetS components including serum triglycerides (TG), plasma glucose, and insulin resistance markers even after adjusting for age, race, body mass index (BMI), smoking status, alcohol consumption, and C-reactive protein (CRP) level [157]. Iron homeostasis is controlled by the iron-regulatory hormone hepcidin and its receptor ferroportin [158]. Dietary iron overload, transfusion-induced iron overload, and inflammatory disorders increase hepcidin. An association exists between high ferritin levels and hepcidin, TNF-α, IL-6, and CRP, which may link iron to T2D. Iron can increase the expression of pro-inflammatory cytokines like TNF-α and IL-6, as well as CRP [159]. CRP concentration is a well-established insulin resistance marker associated with T2D [160], while elevated levels of pro-inflammatory cytokines are biomarkers of obesity [159]. IL-6 also stimulates hepcidin synthesis in hepatocytes [159]. Some evidence suggests that elevated ferritin causes diabetes, although the reverse scenario is also supported [57]. As reviewed, altered iron concentration affects glucose metabolism by generating ROS and disrupting intracellular signaling pathways. High iron-induced oxidative damage reduces insulin gene expression and causes β cell failure and insulin resistance by inhibiting antioxidant defenses like catalase and superoxide dismutase 2. Iron overload reduces HIF-α, which downregulates GLUT1 and GLUT2 transporters, impairing glucose sensing and insulin secretion. However, normal physiological responses may involve ROS generated by intermediate iron levels [57]. Adiponectin levels, which are reduced in obesity, T2D, and CVDs, are negatively correlated with ferritin levels [161]. Adiponectin activates AMPK to increase fatty acid oxidation and glucose uptake in muscle and adipose tissue. AMPK activation promotes glucose uptake by increasing GLUT4 translocation [162]. Although, iron can activate the AMPK pathway independently of adiponectin that generally exerts antidiabetic effects [57]. Iron, therefore, has a complex association with diabetes.

#### 3.2.5 Effects of other heavy metals

Studies link mercury to the pathogenesis of MetS. Mercury-induced oxidative stress causes insulin resistance, hypertension, dyslipidemia, and obesity, however direct association with diabetes is inconclusive [65]. MeHg can modulate the PI3K/AKT signaling pathway, although the manner of modulation is debatable. Some studies have found that MeHg (100 nM to 1 µM for 24 hours) decreases AKT phosphorylation and downregulates PI3K/AKT in neuronal cells [163], while others reported low dose MeHg-induced upregulation of AKT phosphorylation and activation of PI3K/AKT signaling pathway (1 µM to up to 2 µM) through mercury-induced oxidative stress leading to pancreatic β-cell dysfunction associated with diabetes as well as in neuroblastoma cells [163], [164]. The prevalence of hyperglycemia, hypertension, and T2D is also associated with increased nickel exposure [72], [165]. Nickel-induced ROS may damage insulin function and induce glucose deregulation [72].

Similar to chromium, vanadium compounds can enhance insulin response in T2D [166]. Vanadate is a phosphate structural analog that inhibits phosphatases and related enzymes. In diabetic rats, vanadium compounds were found to inhibit multiple phosphatases, especially phosphotyrosine phosphatase that dephosphorylates the autophosphorylated active insulin receptor, suggesting a potential use of vanadium supplements in the treatment of T2D [167]. By preventing the dephosphorylation of tyrosine phosphorylated residues in the insulin receptor, vanadium activates the PI3K/AKT pathway responsible for carbohydrate metabolism. In addition, vanadium also elevates the expression of GLUT4, stimulates glycogenesis, and inhibits glycogenolysis and gluconeogenesis [168]. Table 3 shows the key signaling pathways associated with glucose metabolism and diabetes that are affected by heavy metals and metalloids.

**Table 3:** Major glucose metabolism and diabetes-associated signaling pathways affected by heavy metals and metalloids.

Heavy metals and metalloids	Major signaling pathways associated with glucose metabolism and diabetes
Arsenic	• PI3K/AKT [33], [141] • PPARγ [138] • p38 MAPK [141] • NF-κB/PTEN [139] • Pentose Phosphate Pathway [139] • Pentose/Glucuronate Interconversion [143], [144] • Pro-inflammatory cytokines [139]
Cadmium	• PPARγ [149] • C/EBPα [149] • NF-κB/PTEN [152] • Gluconeogenesis [41], [148] • Glycogenolysis [169] • Pro-inflammatory cytokines [152]
Chromium	• PI3K/AKT [156] • AMPK [156] • JNK MAPK [156] • P-Tyr Phosphatase Inhibition [153]
Iron	• HIF-1α [57] • AMPK [57] • Pro-inflammatory cytokines [159]
Mercury	• PI3K/AKT [163], [164]
Nickel	• Gluconeogenesis [72] • Glycogenolysis [72]
Vanadium	• P-Tyr Phosphatase Inhibition [167]

### 3.3 Lipid metabolism, adipogenesis, and atherosclerosis

#### 3.3.1 Effects of arsenic

Arsenic has an enormous impact on lipid and glycolipid metabolism. Long-term arsenic exposure can deteriorate the structural integrity and functions of the cardiovascular system, leading to CVDs, hypertension, dyslipidemia, obesity, and fatty liver disease [170], [171], [172]. Depending on arsenic species, dose, and affected tissue, subchronic exposure causes distinct dyslipidemia patterns [172]. Arsenic exposure has been linked to an increase in lysoPCs and lipid oxidation, including glycolipids, phospholipids, and cholesterol, as well as alterations in TG and plasma cholesterol [170], [171]. Arsenic-induced oxidative stress damages cells by breaking down membrane phospholipids [170]. Arsenic exposure alters several key polyunsaturated fatty acids (PUFAs) in the gut microbiome [173]. Arsenic exposure is inversely related to dietary lipid (monounsaturated, polyunsaturated, and saturated) intake [174]. Arsenic-induced AKT activation in preadipocytes inhibits preadipocyte differentiation. ATO (at a concentration of 3 µM) promotes AKT expression and phosphorylation and inhibits its interaction with PPARγ [175]. Arsenic-mediated down-regulation of PPARγ also inhibits adipogenic differentiation [176]. Arsenic also disrupts mitochondrial β-oxidation of fatty acids, the principal fat metabolism pathway for energy generation, by inhibiting thiolase leading to partial inhibition of fatty acid oxidation and ketogenesis [177].

Atherogenesis is a pathophysiological condition characterized by inflammation and proliferation of smooth muscle cells, followed by thrombosis and vascular wall damage [27], [178]. Arsenic may cause atherosclerosis by increasing transcription of growth factors like granulocyte-macrophage colony-stimulating factor (GM-CSF) and VEGF, inflammatory cytokines like TNF-α, IL-1 and IL-8, chemokines like monocyte chemoattractant protein-1 (MCP-1), and adhesion molecules such as vascular cell adhesion molecule 1 (VCAM-1), intercellular Adhesion Molecule 1 (ICAM-1) [179], [180]. Pro-inflammatory cytokines and chemokines, such as MCP-1 and IL-6, are elevated in atherosclerotic lesions, the expression of which is also induced by arsenic-generated ROS, suggesting the role of arsenic-induced inflammation in atherosclerosis development [181]. IL-6 plays a key role in the synthesis of acute phase proteins, including CRP [182]. CRP has a major contribution to the development of atherosclerosis. CRP decreases the synthesis of inducible nitric oxide synthase (iNOS) that is responsible for NO imbalance. It also induces the expression of VCAM, ICAM, E-selectin, and MCP-1, and upregulates IL-8 that promotes the recruitment of mononuclear cells in sites of inflammation. Both of these events contribute to atherogenesis [183]. Atherogenesis is strongly associated with an oxidative stress. This process begins with low-density lipoprotein (LDL) oxidation followed by foam cell formation [179]. Generated ROS can act as signal molecules for increased transcription of NF-κB and AP-1, which upregulates vascular adhesion molecules and chemokines such as VCAM-1, MCP-1, TNF-α, IL-1β, and IL-8 [27], [66]. Increased expression of IL-8, NF-κB, and AP-1 can aggravate atherosclerosis by increasing platelet aggregation [27]. Exposure to arsenic and arsenic-induced ROS activates the NF-κB pathway and induces IL-8 expression [66].

Arsenic reduces cyclic guanosine monophosphate (cGMP)—a NO surrogate, and inhibits endothelial nitric oxide synthase (eNOS) in endothelial cells [184]. NO suppresses pro-inflammatory mediators by inactivating the NF-κB pathway. Arsenic also inhibits iNOS by binding to NF-κB and lipopolysaccharide-induced NO production by inactivating NF-κB and ERK1/2 MAPK pathways [185]. Thus, arsenic-mediated reduced NO levels contribute to arsenic-related atherosclerosis and hypertension [184]. Arsenic exposure, however, has been reported to produce NO too. The difference in NO production may be caused by differences in arsenic types, exposure length, and affected cells or tissues [185]. Arsenic inhibits the liver X receptors, which also promotes the risk of atherosclerosis and plaque formation and is supposedly involved in arsenic-induced CVDs [186].

#### 3.3.2 Effects of cadmium

Cadmium exposure is associated with intracellular lipid accumulation and elevated levels of pro-inflammatory lipids, e.g*.*, lysoPCs [152]. Owing to its similarity to zinc, cadmium may displace zinc in antioxidant enzymes like paraoxonase 1, catalase, superoxide dismutase, and glutathione peroxidase. Low levels of paraoxonase 1 activity may be associated with an increased prevalence of CVDs [187]. Cadmium-induced inactivation of antioxidant enzymes also increases lipid peroxidation. Such atherogenic changes in lipid profile increases the incidence of CVDs, stroke, and peripheral artery disease [97]. A dose-dependent increase in LDL and oxidized LDL is associated with cadmium exposure (2 to 50 mg/L CdCl_2_) [188]. An increase in CRP and fibrinogen in population studies and VCAM-1 in animal study, and a decrease in NO have also been reported, similar to arsenic [10]. The impaired NO functioning and signaling is caused by cadmium-induced reduction of phosphorylation of eNOS that causes abnormalities in normal arterial tone [187]. Low dose cadmium treatment is accompanied by an increase in VEGF and upregulation of MAPK (p38, ERK and JNK) pathways [189], and the role of VEGF in human coronary atherosclerosis is well established [190]. Lab studies link cadmium exposure to higher prevalence and mortality from CVDs [188].

#### 3.3.3 Effects of mercury

Mercury, especially MeHg, has a high affinity for —SH groups and selenium, which reduces antioxidant defense by disrupting GSH, GPX, and catalase and promotes free radical-mediated oxidative stress and lipid peroxidation [66], [191] It can promote atherosclerosis by inhibiting NF-κB activation by lipid peroxidation or by binding to the —SH groups present in NF-κB. Inhibition of NF-κB is associated with inactivation of iNOS and suppression of NO production [66]. Consequently, the risk of myocardial infarction and the mortality rate from coronary heart disease and CVDs are increased [191]. Mercury exposure activates p38 MAPK and increases the expression of TNF-α and interferon gamma (IFN-γ), which promote atherosclerosis [192], [193]. Exposure to mercury (concentrations ≥ 10 µM after 24 hours were found to be toxic) can also change membrane K^+^ conductance, modulate chlorine channels, deactivate Na^+^/K^+^-ATPase, inhibit phospholipid turnover, and activate phospholipase C (PLC). However, lower concentrations (0.5 to 5 µM) is enough to cause DNA damage [132], [194].

#### 3.3.4 Effects of nickel

Nickel increases the fluidity of membranes containing phosphatidylinositol-(4,5)-bisphosphate [PI(4,5)P2] and the lipid clustering of phosphatidylinositol-3-phosphate (PI3P) systems, interfering with the development of signaling lipid domains and leading to nickel toxicity [195]. Nickel exposure upregulates the expression of pro-inflammatory cytokines (TNF-α and IL-6), VCAM-1, MCP-1, and cluster of differentiation 68 (CD68). In early atherosclerosis, MCP-1 promotes monocyte adherence. CD68 is a biomarker of macrophage infiltration during atherosclerosis. VCAM-1 promotes monocyte adhesion and accumulation on vessel walls. They provide a plausible mechanism for the increased risk of atherosclerosis associated with nickel [196]. Excess nickel exposure-associated VEGF production in a dose-dependent manner (at concentrations of 125, 250, and 500 µM of NiCl_2_) through AKT, ERK, and NF-κB activation may be analogous to cadmium-associated VEGF production and atherosclerosis as shown in cell culture studies [131], [188].

#### 3.3.5 Effects of other heavy metals

Ferroptosis is an iron-dependent cell death process resulting from iron accumulation and lethal lipid species, e.g*.* ceramide and lysoPC, derived from lipid peroxidation, notably of PUFAs [197], [198]. These lipids and iron-induced ROS inhibit glutathione biosynthesis and glutathione peroxidase, which suppress ferroptosis [119]. Once formed, lipid peroxides enhance ROS signaling and lead to toxic byproduct accumulation (malondialdehydes and 4-hydroxynonenal) that react with DNA bases, proteins, and other nucleophilic molecules, causing cytotoxicity [198]. Chronic iron overload increases both systemic and vascular ROS production, reducing NO bioavailability, impairing vasorelaxation, and accelerating arterial thrombosis [58].

Vanadium can act as a cardioprotective agent. The activation of vanadium-induced PI3K/AKT signaling pathway results in the phosphorylation of eNOS and production of NO. NO, in turn, activates cGMP which leads to the subsequent inhibition of Ca^2+^-ATPase and activation of potassium channels. These cellular events are involved in the regulation of angiogenesis and vasorelaxation [168].

### 3.4 Neurodegeneration

#### 3.4.1 Effects of arsenic

Arsenic-induced neurotoxicity can occur through a number of mechanisms, including ROS-induced oxidative stress, decreased activities of mitochondrial complex I, II-III, and IV, lipid peroxidation followed by DNA damage and neuronal cell death, apoptosis by caspase-9, p38, and JNK activation, decreased acetylcholinesterase activity, and Ca^2+^ imbalance. These are linked to central and peripheral nervous system damage and may explain arsenic-related neurological symptoms [199], [200], [201], [202]. There is a dose- (5 to 150 ppb) and exposure (acute vs. chronic) dependent relationship between higher arsenic levels in drinking water and peripheral nerve abnormalities [11]. Neurological impacts of arsenic include depression, insomnia, anxiety, and cognitive impairments affecting vocabulary, mental acuity, language precision, IQ, and comprehension [203]. As[III] generates β-amyloid plaques and hyperphosphorylated tau proteins—pathological hallmarks of Alzheimer's disease [204]. Arsenic reduces stem cell development into neurons by altering the feedback loop between WNT and Notch signaling pathways [205].

#### 3.4.2 Effects of iron

Since the brain consumes 20% of body oxygen, the central nervous system (CNS) is especially vulnerable to oxidative stress. Iron overexposure causes oxidative stress and ROS, which upregulate the *c-fos* gene. The Fos transcription regulator forms a complex, AP-1, with another protein named Jun. The AP-1 site is in the promoter region of several neuronal activity or degeneration genes. Iron-induced oxidative stress may activate early genes, which may explain the link between iron toxicity and neurological disorders such as epilepsy, stroke, Alzheimer’s disease, Parkinson's disease, Huntington’s disease, and amyotrophic lateral sclerosis (ALS) [60]. Iron-induced ferroptosis may contribute to Alzheimer's, Parkinson's, and Huntington's disease [206], [207], [208]. Iron accumulation in glial cells may promote neuroinflammation and aging [209]. Accumulation of iron in the brain is also connected with aceruloplasminemia and neuroferritonopathy [11]. Free iron-induced oxidative damage also activates the PI3K/AKT pathway which inactivates glycogen synthase kinase 3-beta (GSK3β). GSK3β plays a key role in the pathogenesis of Alzheimer's disease, Huntington’s disease, and bipolar disorder [210].

#### 3.4.3 Effects of mercury

The CNS is the primary target of MeHg toxicity. Mercury-induced alterations in protein synthesis, a key factor in cellular degeneration, may cause nervous system changes [211]. Another important factor in MeHg-induced neurotoxicity is oxidative stress that damages mitochondria. MeHg accumulation and oxygen depletion alter electron transport and mitochondrial membrane potential, inducing apoptosis [132]. Furthermore, MeHg inhibits astrocytic HIF-1α and related downstream genes like GLUT1 and VEGF in a time- and concentration-dependent manner, resulting in lower cell proliferation and higher cytotoxicity in primary rat astrocytes [212]. AKT phosphorylation is similarly lowered by MeHg exposure (1 µM for 24 hours) in neuronal cells, resulting in downregulation of the PI3K/AKT signaling pathway that triggers caspase-3-dependent apoptosis and lowers neuronal viability [163]. Non-canonical Notch signaling pathways affect the developing drosophila fetal nervous system [213]. Mercury exposure increases the chance of neurological diseases like Alzheimer's. Alzheimer's disease patients have higher brain mercury levels than normal individuals [132]. Mercury inhibits guanosine triphosphate (GTP) binding, even at low concentrations, which is required for tubulin synthesis and neuronal function [214]. Case reports also suggest a link between inorganic mercury exposure and Alzheimer’s disease, multiple sclerosis, and ALS [11], [215].

#### 3.4.4 Effects of vanadium

Vanadium-induced neuropathology, including neurobehavioral, neurochemical, and neurocellular changes, is known [82]. Vanadium-induced apoptosis and DNA cleavage leading to neuroinflammation, disruption of the blood brain barrier, dendritic spine loss, and behavioral, cognitive, and motor impairments including memory alteration are due to ROS generation and consequent lipid peroxidation [216]. Vanadate induces both extrinsic and intrinsic apoptosis in cell cultures [124]. Chronic vanadium exposure activates microglial cells which is associated with neurodegenerative conditions like Alzheimer's disease [82].

Interestingly, protective effects of vanadium compounds on cognitive impairments and Alzheimer's are also known. A link exists between T2D and Alzheimer's disease. Amyloid β binds to insulin receptor and alters downstream AKT and GSK3β activity, impairing brain insulin signaling [217], [218], [219]. Additionally, tau deletion induces brain insulin resistance through altered IRS-1 and phosphatase and tensin homolog deleted on chromosome ten (PTEN) activities, leading to cognitive and metabolic impairments [220]. While vanadium compounds are strong potential candidates for treating T2D [221], [222], one improves the pathological alterations involved in Alzheimer's disease [223]. This compound, known as BEOV (bis—(ethylmaltolato)oxidovanadium), increases PPARγ causing amyloid β downregulation and inhibits tau hyperphosphorylation by modulating the AKT/GSK3β pathway. Another vanadium-containing enzyme that mimics around six naturally occurring antioxidants (vanadium carbide MXene-based nanoenzyme) can reestablish redox homeostasis and ameliorate oxidative stress-induced neurodegenerative and inflammatory disorders [28].

#### 3.4.5 Effects of other heavy metals

Cadmium-induced apoptosis is linked to ROS generation, Ca^2+^ accumulation, caspase-3 upregulation, B-cell lymphoma 2 (BCL-2) downregulation, and p53 deficiency [97]. Cadmium-generated ROS induces neuronal apoptosis by activating mTOR and PI3K/AKT pathways while inhibiting PTEN and AMPK pathways [224]. Tau protein buildup, which is linked to Alzheimer's disease, is accelerated by cadmium [203]. Cadmium also promotes neuronal cell death by activating the Fas-Fas ligand (FasL) pathway, which may explain its neurotoxicity [225].

Limited literature exists on chromium’s neurotoxicity. The role of chromium-generated ROS in the brain is unknown. One study reported that Cr[VI] elevated ICAM-1 and VCAM-1 expression and activated NF-κB–caspase-1–IL-1β cascade which may associate chromium with neurodegenerative diseases [226].

Nickel increases the expression of caspases, cytochrome c, BCL-2-associated X protein (BAX), and BH3-interacting domain death agonist (BID) proteins, whereby lowering the expression of BCL-2. BAX and BID proteins are proapoptotic, whereas BCL-2 is antiapoptotic [227]. These proteins initiate intrinsic apoptosis by releasing mitochondrial cytochrome c and activating caspase-9 [228]. Nickel also promotes Fas-FasL interaction in the extrinsic apoptotic pathway, triggering caspase-8 and caspase-10 pathways [75]. Excessive apoptosis is responsible for many neurodegenerative diseases and nickel-induced lung inflammation [75]. Table 4 summarizes the predominant extrinsic and intrinsic apoptotic pathways that are associated with heavy metal-induced neurotoxicity.

**Table 4:** Neurotoxicity-associated predominant pathways affected by heavy metals and metalloids.

Heavy metals and metalloids	Principal pathways involved in neurotoxicity	
	**Caspase pathways**	**Others**
Arsenic	• Caspase-9 by cyt c release [199]	• ROS generation [202] • Ca^2+^ imbalance [202] • p38 and JNK [202] • WNT and Notch [205] • Reduced acetylcholinesterase activity [201]
Cadmium	• Caspase-9 by p53 deficiency & reduced BCL-2 [97] • Caspase-8 by Fas-FasL [225]	• ROS generation [97] • Ca^2+^ accumulation [97] • mTOR & PI3K/AKT [224] • PTEN & AMPK [224]
Chromium	• Caspase-1 by NF-κB [226]	
Iron		• Ferroptosis [229] • AP-1 [60] • PI3K/AKT [210]
Mercury	• Caspase-3 by PI3K/AKT [163]	• HIF-1α [212]
Nickel	• Caspase-9 by BAX, BID, and cyt c [227] • Caspase-8 and caspase-10 by Fas-FasL [75]	
Vanadium	• Caspase-9 by cyt c release [124] • Caspase-8 by Fas-FasL [124]	

### 3.5 Inflammation and immune response

#### 3.5.1 Effects of arsenic

Arsenic exposure alters macrophage, dendritic, and T lymphocyte development, activation, and/or proliferation [230]. Chronic arsenic exposure inhibits NF-κB-related survival pathways and increases caspase-3 and caspase-8 activity, leading to monocytic apoptosis [231]. In addition, monocyte-derived macrophages are accompanied with reduced adhesion capacity, decreased NO production, diminished CD54 and F-actin expression, and impaired phagocytic activity and macrophage functions [232]. At higher concentrations, arsenic decreases the phagocytic activity of dendritic cells and dendritic cell-dependent T cell activation. Human CD4^+^ and CD8^+^ T cells are apoptosed by arsenic-induced ROS [230]. Arsenic exposure increases neutrophil apoptosis through MAPKs. Treatment with iAs decreases phagocytic activity and increases Toll-like receptors TLR2 and TLR4 production, modulating host immune response and causing adverse effects [233]. Pro-inflammatory cytokines, growth factors, and chemokines, such as TNF-α, IL-1β, IL-6, IL-8, IL-12, CRP, and MCP-1, are upregulated in individuals exposed to arsenic for a long time [230], [234], [235]. TNF-α is upregulated in chronic low level arsenic exposure that may contribute to the systemic inflammation through TNF signaling-mediated apoptosis of CD4^+^ T cells [234], [236]. This phenomenon, known as arsenic immunotoxicity, characterized by limited immune surveillance evident from decreased bacterial phagocytosis by macrophages and reduced T cell proliferation, increases susceptibility to infections. Arsenic-induced immunosuppression is associated with an increased incidence of diarrhea, respiratory tract infections such as influenza A and pulmonary tuberculosis, and even lung cancer [230]. However, arsenic-induced immunosuppression may prevent or treat immune system damage in related diseases.

Inflammasome complexes are composed of NOD-like receptor proteins (NLRs) that specifically control caspase-1-dependent cleavage of pro-IL-1β and pro-IL-18. Arsenite and ATO suppress pro-IL-1β cleavage by inhibiting NLRP1, NLRP3, and NAIP5/NLRC4 [230]. The liver plays a crucial role in the immune complex-mediated removal of foreign antigens with the help of IgG mediated by Fcγ receptor (FcγR) binding [237]. Low level chronic arsenic exposure suppresses the expression of FcγR and complement receptors [234].

#### 3.5.2 Effects of cadmium

Cadmium impairs both innate and adaptive immunity. As reviewed, cadmium exposure impairs macrophage surface FcγRII expression and phagocytic capacity, inhibits macrophage activity in response to lipopolysaccharides and TLRs via decreased production of pro-inflammatory cytokines such as TNF-α and IL-1, induces immunosuppressive reactions and apoptosis of neutrophils and dendritic cells, and reduces the number of natural killer (NK) cells in the field of innate immunity. In case of adaptive immunity, cadmium decreases the CD4^+^/CD8^+^ ratio, downregulates cytokine production in Th1 (e.g*.*, IFN-γ and IL-2) and Th2 (e.g*.*, IL-4) lymphocytes, suppresses the expression of class I and class II major histocompatibility complex (MHC) molecules in B lymphocytes, alters signaling through them, and inhibits B-lymphocytic activity and immunoglobulin IgE synthesis [238]. Short-term cadmium exposure (10000 ppb for 4 weeks) can provoke an immunological reaction that leads to autoimmune disorders including proteinuria in murine models [239]. The authors also found that chronic low-dose cadmium exposure increases IgG2a synthesis, which can cause autoimmune glomerulonephritis.

Cadmium causes respiratory disorders. Cadmium-induced oxidative stress enhances influenza virus proliferation. Cadmium-induced redox imbalance and subsequent activation of redox-sensitive cascades, including protein kinase C (PKC) and p38, JNK, and ERK MAPK pathways, may contribute to the process [240]. Higher cadmium levels increase mortality from influenza and pneumonia and may aggravate COVID-19 pulmonary consequences [241]. Tight junctions control selective paracellular diffusion of ions and solutes at bodily compartment boundaries [242]. Several protein kinases including proto-oncogene tyrosine-protein kinase (c-Src) and PKC modulate tight junction function and integrity. Cadmium exposure collapses this barrier function and increases chemical and biomolecule penetration by activating PKC or directly interrupting junctional interacting protein genes [243]. Ras-related protein 1 (Rap1) signaling is also involved in tight junction formation with atypical PKC [244]. In human airway edema cells, cadmium elevates mucin 8 expression through TLR4 mediated activation of ERK1/2 and p38 MAPK pathways [245]. Increased levels of mucin are positively associated with morbidity and mortality in inflammatory airway diseases such as chronic bronchitis, asthma, and chronic obstructive pulmonary disease (COPD) [246].

#### 3.5.3 Effects of mercury

Both organic and inorganic forms of mercury are immunotoxins [247]. Mercury induces autoimmune disease by altering cytokine levels. It triggers Th2 cytokines like IL-4 and suppresses Th1 cytokines like IFN-γ. Higher amounts of mercuric chloride can activate PKC that plays an important role in IL-4 production. On the other hand, MeHg is known to induce apoptosis in T lymphocytes which may explain the decrease in IFN-γ [248]. Mercuric chloride has a gender-specific immunotoxic effect on cytokine production (IL-2, IL-4, IL-10, and IFN-γ) in adult mice—inhibitory in females, stimulatory in males [249]. Immunotoxic effects of mercury can increase susceptibility to infections, malaria, and immunologically-mediated diseases [132]. Proteinuria, nephrotic syndrome, and membranous glomerulopathy are mercury-induced autoimmune renal consequences [247]. Mercury alters the estrogenic effects on thymus development [70]. Inorganic mercury impairs the immune system, causing Kawasaki-like symptoms [250].

#### 3.5.4 Effects of other heavy metals

Immune response to Cr[VI] is dose-dependent. Lower concentrations of Cr[VI] stimulate lymphocyte blastogenesis, whereas higher concentrations inhibit it [251]. Likewise, lower doses of Cr[VI] enhance humoral immune responses and macrophage phagocytic activity, but higher doses reduce any such effect [252]. Cr[VI] exposure is associated with rhinitis, bronchospasm, bronchial asthma, and pneumonia as well as impaired respiratory dynamics [253]. Chromium-induced ROS activates NF-κB, a key gene activator in inflammation, immunity, and apoptosis. Studies also link chromium to autoimmune disorders and hypersensitivity [253], [254].

Iron causes hepatic inflammation. Iron overload causes oxidative stress, which activates apoptotic pathways in hepatocytes via Fas-FasL or TNF-TNF receptors, in individuals with chronic hepatitis C [62]. In hepatic fibrosis, oxidative stress reduces hepcidin expression. This is because the hepcidin transcription factor CCAAT/enhancer binding protein (C/EBP) cannot bind to the hepcidin promoter region as binding is inhibited by C/EBP homology protein expression, which is positively associated with ROS production [255]. Activation of histone deacetylase by ROS also affects the C/EBP binding capacity to the hepcidin promoter [256]. Hepatic iron buildup is also linked to chronic hepatitis B, alcoholic liver disease, and non-alcoholic fatty liver disease [62].

Vanadium compounds are both inflammatory and immunosuppressors [257]. Vanadate induces pro-inflammatory cytokines, such as IL-6, IL-8, and TNF-α, that contribute to the vanadium-induced respiratory inflammation [[258], [259]. Vanadium pentoxide (V_2_O_5_) affects IL-2-dependent PI3K/AKT/mTOR and MAPK pathways. It has also been reported to dysregulate or inhibit humoral responses in human and mice [257]. Inorganic vanadium compounds are known to activate the NF-κB pathway, which regulates the expression of pro-inflammatory mediators among other genes. However, a direct link between NF-κB pathway activation and vanadium-induced inflammatory responses is not yet known [124].

### 3.6 Metal allergy

Metal hypersensitivity is a rising concern with around 10-15% of human population being afflicted by contact hypersensitivity [260]. Allergic responses to chromium, nickel, and mercury are the best-studied of all heavy metals. Chromium induces both type I (anaphylactic type) and type IV (delayed-type) hypersensitivity reactions [254]. After exposure, a person with a family history of atopy is more likely to develop a chromium allergy. Covalently bound chromium (Cr[III]) compounds with —SH groups, DNA-chromium-protein cross-linked compounds, or oxidized proteins may act as allergenic epitopes [253]. Hexavalent chromium compounds are also known to cause systemic contact dermatitis [261]. In one study, chromium exposure from Co-Cr hip prostheses increased TNF-α, GM-CSF, and IL-6 [262]. ROS induced by Cr[VI] causes K^+^ efflux and NLRP3 activation [263]. NLRP3 activates caspase-1, which cleaves the precursors of the allergic cytokines IL-1β and IL-18 [264]. Occupational exposure to chromium and its compounds has been reported to cause perforations of the nasal septum, bronchial asthma, allergic rhinitis, and contact allergic eczema [254].

Detailed nickel allergy mechanism is unknown, however the skin inflammation reaction involves production of cytokines and chemokines, activation of antigen presenting cells that present nickel allergen to naive CD4^+^ T cells, and activation and proliferation of T cells following re-exposure [75]. According to a recent study, nickel directly activates human TLR4. Nickel-induced TLR4 activation leads to further activation of NF-κB and p38, inducing multiple pro-inflammatory cytokines that trigger an allergic response. In this way, nickel directly triggers NF-κB-dependent activation of human dendritic cells, whereas other contact allergens require a second stimulus [265]. According to a study on systemic nickel allergy, there is a dose-relationship between the amount of nickel ingested and the frequency of dermatitis flare-ups, with even a single dose of 4 mg of nickel causing widespread dermatitis in most nickel-allergic patients. Therefore, it is recommended that such individuals follow a low-nickel diet [261].

Mercury exposure is known to cause both type I (anaphylactic type) and type IV (delayed-type) hypersensitivity [266]. The most common allergic event is dental amalgam-related mercury allergy. Oral lichen planus, acrodynia, Kawasaki disease, and tattoo allergy are notable among others caused by prolonged exposure to mercuric compounds [250], [267]. Local cytotoxic injury and subsequent tissue damage, i.e., injury of epithelial cells by T lymphocytes, is the most common mechanism of allergic response to mercury. Lyphadenopathy, transport of metal particles via the lymphatic system from skin or tissue followed by phagocytosis in macrophages, is another delayed-type (type IV) hypersensitivity reaction to mercury [267].

## 4 Conclusion

Heavy metal pollution is a global health and environmental concern. Sufficient evidence has supported the designation of ambient metal pollutants to be regarded as risk factors for cardiovascular, respiratory, metabolic, and neurological disorders. Rapid, uncontrolled urbanization and industrialization have exacerbated the release and exposure of heavy metals. Essential trace metals are needed for normal cellular and enzyme activity, including cellular metabolism, protein folding, DNA replication, redox reactions, immune reactions, electron transport and other metabolite transport, signal transduction, and neurotransmitter synthesis. However, high levels of these and other non-essential heavy metals and metalloids can damage biomolecules, impair cellular redox status, alter signal transduction, and cause protein misfolding, apoptosis, and malignant transformation.

We reviewed the association between arsenic, cadmium, chromium, iron, mercury, nickel, and vanadium exposure and disruptions in common metabolic signaling pathways and pathophysiological conditions such as diabetes, carcinogenesis, cardiovascular, neurodegenerative, allergic, and inflammatory diseases. Among these are the MAPK, AMPK, PI3K/AKT, NF-κβ, HIF-1, TNF-α, TLR, NLR, WNT/β-catenin, Notch, TGF-β/BMP, ferroptosis, and caspase signaling pathways associated with such toxicities. Almost all the heavy metals lead to an imbalance in the homeostasis of cellular antioxidants (Supplementary table 1).

Some of these metals actually play a protective role by targeting those intracellular metabolic pathways. Arsenic and iron have anti-cancer properties, whilst chromium and vanadium supplements are now being investigated and trialled for the treatment of type 2 diabetes. Despite its possible involvement in carcinogenicity and neurotoxicity, vanadium is a pretty interesting metal since its complexes display potent insulin-mimetic effects that have promoted its use as an adjuvant in treating type 2 diabetes, and the ability to ameliorate the pathological conditions that are altered in neurodegenerative Alzheimer’s disease.

The association between pathological states and alterations in metabolic pathways is complex, intricate, and dynamic in nature. This is a brief summary of the As, Cd, Cr, Fe, Hg, Ni, and V affected pathways and some associated diseases that are still being studied. Details and other inconclusive correlations between these metals and other diseases, such as renal toxicity, osteoporosis, gastrointestinal problems, etc., are not mentioned here.

## Funding

Grant for Advanced Research in Education (GARE) from the Ministry of Education, Bangladesh.

## Declaration of Competing Interest

The authors declare that they have no known competing financial interests or personal relationships that could have appeared to influence the work reported in this paper.

## Data Availability

Data will be made available on request.
