# Supplementary material for: Disease-associated metabolic pathways affected by heavy metals and metalloid
Source: Toxicol Rep. 2023 Apr 24;10:554–70. doi: 10.1016/j.toxrep.2023.04.010 (PMC10313886; doi:10.1016/j.toxrep.2023.04.010)
Supplement: Supplementary file 1 — Supplementary material. [file mmc1.doc]

**Supplementary table 1:** Prime targets of the antioxidant defense and protein metabolism by heavy metals and metalloids.

| **Heavy metals and metalloids** | **Targets of imbalance in antioxidant defense and protein metabolism** | |
| --- | --- | --- |
|  | **Antioxidants** | **Others** |
| Arsenic | - Glutathione [1] | - Uroporphyrinogen decarboxylase [2] - Electron transport chain complexes [3] - Pyruvate dehydrogenase [4] - α-ketoglutarate dehydrogenase [5] - Pyruvate carboxylase [6] - Glucose-6-phosphate dehydrogenase [7] - Alkaline phosphatase [8] - Aspartate transaminase [8] - Alanine transaminase [8] - Lactate dehydrogenase [9] |
| Cadmium | - Glutathione [10] - Superoxide dismutase [10] - Catalase [10] | - Electron transport chain complex III [11] - Mitochondrial proteins and transporters [12] - Pyruvate carboxylase [13] - Phosphoenolpyruvate carboxykinase [13] - Fructose 1,6-bisphosphatase [13] - Glucose-6-phosphatase [13] - ATPase [10] - Lactate dehydrogenase [10] - Glutathione peroxidase [10] |
| Chromium | - Glutathione [14] | - Glutathione reductase [14] - Cytochrome P450 [14] - Hemoglobin [14] |
| Iron | - Glutathione [15] | - GPX4 [15] |
| Mercury | - Glutathione [16] - Superoxide dismutase [16] - Catalase [16] | - Glutathione peroxidase [17] - Thioredoxin reductase [17] - Na^+^/K^+^ ATPase [17] - Phospholipase C [18] |
| Nickel | - Glutathione [19] - Superoxide dismutase [19] - Catalase [19] | - Zn-carboxypeptidase [20] - Co-chelatase [20] - Nitrous oxide reductase [20] |
| Vanadium | - Glutathione [4] | - ATPases (Na^+^/K^+^ ATPase, K^+^ ATPase, Ca^2+^ ATPase etc.) [4] - Phosphatases (glucose-6-phosphatase, alkaline phosphatase, acid phosphatase etc.) [4] - Ribonuclease [4] - Phosphodiesterase [4] - Tyrosine kinase phosphorylase [4] - Adenylate cyclase [4] - NADPH oxidase [4] |

[1] R. Hubaux, D.D. Becker-Santos, K.S.S. Enfield, D. Rowbotham, S. Lam, W.L. Lam, V.D. Martinez, Molecular features in arsenic-induced lung tumors, Mol. Cancer. 12 (2013) 20 1–11. https://doi.org/10.1186/1476-4598-12-20.

[2] P. Apostoli, M. Sarnico, P. Bavazzano, D. Bartoli, Arsenic and porphyrins, Am. J. Ind. Med. 42 (2002) 180–187. https://doi.org/10.1002/ajim.10123.

[3] N. Dwivedi, A. Mehta, A. Yadav, B.K. Binukumar, K.D. Gill, S.J.S. Flora, MiADMSA reverses impaired mitochondrial energy metabolism and neuronal apoptotic cell death after arsenic exposure in rats, Toxicol. Appl. Pharmacol. 256 (2011) 241–248. https://doi.org/10.1016/j.taap.2011.04.004.

[4] J. Briffa, E. Sinagra, R. Blundell, Heavy metal pollution in the environment and their toxicological effects on humans, Heliyon. 6 (2020) e04691. https://doi.org/10.1016/j.heliyon.2020.e04691.

[5] C.H. Tseng, The potential biological mechanisms of arsenic-induced diabetes mellitus, Toxicol. Appl. Pharmacol. 197 (2004) 67–83. https://doi.org/10.1016/J.TAAP.2004.02.009.

[6] L. Szinicz, W. Forth, Effect of As_2_O_3_ on gluconeogenesis, Arch. Toxicol. 61 (1988) 444–449. https://doi.org/10.1007/BF00293690.

[7] A. Kulshrestha, U. Jarouliya, G. Prasad, S. Flora, P.S. Bisen, Arsenic-induced abnormalities in glucose metabolism: Biochemical basis and potential therapeutic and nutritional interventions, World J. Transl. Med. 3 (2014) 96–111. https://doi.org/10.5528/wjtm.v3.i2.96.

[8] K. Islam, A. Haque, R. Karim, A. Fajol, E. Hossain, K.A. Salam, N. Ali, Z.A. Saud, M. Rahman, M. Rahman, R. Karim, P. Sultana, M. Hossain, A.A. Akhand, A. Mandal, H. Miyataka, S. Himeno, K. Hossain, Dose-Response Relationship between Arsenic Exposure and the Serum Enzymes for Liver Function Tests in the Individuals Exposed to Arsenic: A Cross Sectional Study in Bangladesh, Environ. Heal. A Glob. Access Sci. Source. 10 (2011) 64 1–11. https://doi.org/10.1186/1476-069X-10-64.

[9] Y.T. Liao, C.J. Chen, W.F. Li, L.I. Hsu, L.Y. Tsai, Y.L. Huang, C.W. Sun, W.J. Chen, S.L. Wang, Elevated lactate dehydrogenase activity and increased cardiovascular mortality in the arsenic-endemic areas of southwestern Taiwan, Toxicol. Appl. Pharmacol. 262 (2012) 232–237. https://doi.org/10.1016/j.taap.2012.04.028.

[10] G. Genchi, M.S. Sinicropi, G.G. Lauria, A. Carocci, A. Catalano, The Effects of Cadmium Toxicity, Int. J. Environ. Res. Public Health. 17 (2020) 3782 1–24. https://doi.org/10.3390/ijerph17113782.

[11] E.A. Belyaeva, T.V. Sokolova, L.V. Emelyanova, I.O. Zakharova, Mitochondrial Electron Transport Chain in Heavy Metal-Induced Neurotoxicity: Effects of Cadmium, Mercury, and Copper, Sci. World J. 2012 (2012) 136063 1–14. https://doi.org/10.1100/2012/136063.

[12] Y. Wang, J. Fang, S.S. Leonard, K.M.K. Rao, Cadmium inhibits the electron transfer chain and induces reactive oxygen species, Free Radic. Biol. Med. 36 (2004) 1434–1443. https://doi.org/10.1016/j.freeradbiomed.2004.03.010.

[13] Z. Merali, R.L. Singhal, Protective effect of selenium on certain hepatotoxic and pancreotoxic manifestations of subacute cadmium administration., J. Pharmacol. Exp. Ther. 195 (1975) 58–66. http://www.ncbi.nlm.nih.gov/pubmed/171375.

[14] A.D. Dayan, A.J. Paine, Mechanisms of Chromium Toxicity, Carcinogenicity and Allergenicity: Review of the Literature from 1985 to 2000, Hum. Exp. Toxicol. 20 (2001) 439–451. https://doi.org/10.1191/096032701682693062.

[15] J. Li, F. Cao, H. liang Yin, Z. jian Huang, Z. tao Lin, N. Mao, B. Sun, G. Wang, Ferroptosis: past, present and future, Cell Death Dis. 11 (2020) 88. https://doi.org/10.1038/s41419-020-2298-2.

[16] P.A. Olsvik, H. Amlund, B.E. Torstensen, Dietary lipids modulate methylmercury toxicity in Atlantic salmon, Food Chem. Toxicol. 49 (2011) 3258–3271. https://doi.org/10.1016/j.fct.2011.09.025.

[17] A. Carocci, N. Rovito, M.S. Sinicropi, G. Genchi, Mercury Toxicity and Neurodegenerative Effects, in: Reviews of Environmental Contamination and Toxicology, Springer International Publishing, Cham. (2014) 1–18. https://doi.org/10.1007/978-3-319-03777-6.

[18] I. Panfoli, B. Burlando, A. Viarengo, Effects of heavy metals on phospholipase C in gill and digestive gland of the marine mussel Mytilus galloprovincialis Lam, Comp. Biochem. Physiol. - B Biochem. Mol. Biol. 127 (2000) 391–397. https://doi.org/10.1016/S0305-0491(00)00272-8.

[19] G. Liu, L. Sun, A. Pan, M. Zhu, Z. Li, Z. Wang, X. Liu, X. Ye, H. Li, H. Zheng, C.N. Ong, H. Yin, X. Lin, Y. Chen, Nickel exposure is associated with the prevalence of type 2 diabetes in Chinese adults, Int. J. Epidemiol. 44 (2015) 240–248. https://doi.org/10.1093/ije/dyu200.

[20] G. Genchi, A. Carocci, G. Lauria, M.S. Sinicropi, A. Catalano, Nickel: Human health and environmental toxicology, Int. J. Environ. Res. Public Health. 17 (2020) 679. https://doi.org/10.3390/ijerph17030679.
